# Dop1R1, a type 1 dopaminergic receptor expressed in Mushroom Bodies, modulates *Drosophila* larval locomotion

**DOI:** 10.1371/journal.pone.0229671

**Published:** 2020-02-26

**Authors:** Bryon Silva, Sergio Hidalgo, Jorge M. Campusano

**Affiliations:** 1 Laboratorio Neurogenética de la Conducta, Departamento de Biología Celular y Molecular, Facultad de Ciencias Biológicas, Pontificia Universidad Católica de Chile, Santiago, Chile; 2 Genes and Dynamics of Memory Systems, Brain Plasticity Unit, CNRS, ESPCI Paris, PSL Research University, Paris, France; 3 School of Physiology, Pharmacology and Neuroscience, Faculty of Life Science, University of Bristol, Bristol, United Kingdom; 4 Centro Interdisciplinario de Neurociencias, Pontificia Universidad Católica de Chile, Santiago, Chile; Biomedical Sciences Research Center Alexander Fleming, GREECE

## Abstract

As in vertebrates, dopaminergic neural systems are key regulators of motor programs in insects, including the fly *Drosophila melanogaster*. Dopaminergic systems innervate the Mushroom Bodies (MB), an important association area in the insect brain primarily associated to olfactory learning and memory, but that has been also implicated with the execution of motor programs. The main objectives of this work is to assess the idea that dopaminergic systems contribute to the execution of motor programs in *Drosophila* larvae, and then, to evaluate the contribution of specific dopaminergic receptors expressed in MB to these programs. Our results show that animals bearing a mutation in the dopamine transporter show reduced locomotion, while mutants for the dopaminergic biosynthetic enzymes or the dopamine receptor Dop1R1 exhibit increased locomotion. Pan-neuronal expression of an RNAi for the Dop1R1 confirmed these results. Further studies show that animals expressing the RNAi for Dop1R1 in the entire MB neuronal population or only in the MB γ-lobe forming neurons, exhibit an increased motor output, as well. Interestingly, our results also suggest that other dopaminergic receptors do not contribute to larval motor behavior. Thus, our data support the proposition that CNS dopamine systems innervating MB neurons modulate larval locomotion and that Dop1R1 mediates this effect.

## Introduction

Biogenic amines (BAs), among them dopamine, are molecules extensively distributed in the vertebrate central nervous system (CNS), which act on specific receptors to modulate a wide range of behaviors including the execution of motor programs [1, 2]. BAs are also expressed in the CNS of invertebrates including the fly *Drosophila melanogaster* [3] and have been implicated in the generation and execution of motor programs [4, 5]. However, the neural mechanisms underlying the contribution of BA systems to locomotion are far from being completely understood either in vertebrates or invertebrates.

In the adult fly brain, two are the main structures responsible for the generation and modulation of motor programs: the Central Complex (CC) and the Mushroom Bodies (MB), respectively [6–8]. These structures receive strong aminergic innervation, and therefore it is possible to suggest that through the innervation of these structures BA systems exert their actions on motor programs [9–12].

*Drosophila* larvae are also capable to execute motor programs. Importantly, the lower complexity of the larval brain makes it a good system to assess the contribution of specific neural systems to locomotion. Thus, data available suggest that larval motor programs depend on neurons that will become the CC in the adult fly brain and on the larval MB [13, 14]. Moreover, it is possible to propose that as in adult flies, aminergic systems innervating these structures [15, 16] modulate motor output. Actually, we and others have previously shown that serotonin neural systems regulate motor programs, an effect that depend at least partially on specific serotonergic receptors expressed in the larval MB [17–19].

In addition to serotonergic neurons, dopaminergic neural systems also innervate the MB and have been associated to the generation of new olfactory memories in larvae [16]. The possibility that dopaminergic systems that innervate the larval MB modulate the execution of motor programs has not been comprehensively assessed. Here, we advanced on this issue. Several receptors for dopamine have been cloned in *Drosophila* [20–23], but we focused our work on those that share homology with vertebrate DA receptors (Table 1): the D1-type receptors Dop1R1 (aka dDA1; CG9652) and Dop1R2 (aka DAMB; CG18741) and the D2-type receptor Dop2R (aka D2R, DD2R; CG18314).

**Table 1:** Some of the properties of cloned *Drosophila* DA receptors.

BA receptor^a^	Homolog to (vertebrates)	Signaling cascade associated^b^	Gene	Reference
Dop1R1 (dDA1/DmDOP1)	D1-type	Increase cAMP	CG9652	[22]
Dop1R2 (DAMB)	D1-type	Increase cAMP	CG18741	[21]
Dop2R (D2R; DD2R)	D2-type	Decrease cAMP	CG33517	[20]
DopEcR (DmDopEcR)	not applicable (DA-ecdysone receptor)	Increase cAMP Increase PI3K	CG18314	[23]

^a^ Other names used for each receptor are between parentheses.

^b^ Some of the signaling cascades associated to each receptor have been only demonstrated in heterologous systems.

## Materials and methods

### Fly stocks and crosses

Flies were reared in standard food at 19°C in a 12/12 h light/dark cycle. When the Gal4-UAS binary system was used, male flies containing a specific UAS-RNAi element were crossed overnight to virgin female flies containing a Gal4 driver. New animals from F1 were kept at 19°C to diminish the effects of Gal4-driven genes on development [24]. One day before the beginning of an experiment, animals were brought to room temperature (24–25°) to boost the Gal4-driven expression of specific RNAi for the different receptors. The mutant flies used in this work were obtained from the Bloomington Drosophila Stock Center (BDSC, Indiana University, IN, USA), unless indicated otherwise. Flies used were as follows: w^1118^;Dop1R2^MB0518^ (BDSC #24743); w^1118^;Dop2R^f06521^ (Exelixis Collection, Harvard Medical School); y^1^w*;Dop1R1^MI03085^/TM3,Sb^1^Ser^1^ (BDSC #36428); w^1118^;TH(ple)^f01945^/TM6B,Tb^1^ (BDSC # 18492); the cAMP-phosphodiesterase mutant (dnc^1^, BDSC # 6020) and the dopamine plasma membrane transporter (DAT, BDSC #25547) which was cantonized for at least 6 generations. Ddc^ts2^/CyO mutant fly was originally part of Dr Diane O’Dowd Lab fly stock (University of California Irvine, CA, USA). Since mutants have been generated in different genetic backgrounds, different wild-type strains were used as control whenever necessary: Canton-S, y^1^v^1^ and w^1118^. Gal4 driver strains were used according to previous expression data [25] (Table 2). The UAS-RNAi lines directed to the different dopamine receptors were: y^1^v^1^;UAS-RNAi^Dop1R2^ (BDSC; line #26018); y^1^v^1^;UAS-RNAi^Dop1R1^ (BDSC; line #31765); y^1^v^1^;UAS-RNAi^Dop2R^ (BDSC; line #26001). These RNAi and mutant lines have been previously validated elsewhere [26–28]. Data was obtained from animals generated from at least four independent crosses of parental strains, which were maintained in individual vials.

**Table 2:** Expression pattern of the MB Gal4 drivers used in this work.

Gal4 lines	Larval MB expression	
	γ lobe	α′β′ lobe
OK107-Gal4	+	+
MB247-Gal4	+	
201y-Gal4	+	
c305a-Gal4		+

Contribution of drivers to the two larval MB lobes is indicated. Information as previously stated [25].

### Video tracking

As previously described [17]. Briefly, a single third instar larva at the middle of the foraging stage was placed in the center of a 35 mm petri dish half-filled with 1% agar. Larva movement was recorded for 140 secs (Olympus Digital Camera) in a closed box to avoid influence of external stimuli. Locomotion in larvae is a behavior that can be understood as composed by several components: actual movement, pauses and head-sweeps. Larvae at the developmental stage used in this work and under our experimental conditions, are constantly moving. Therefore, we studied the distance covered by the animal (in mm) as a representation of motor output, as previously discussed [17]. To do that, videos were analyzed using an automated tracking system (Image-Pro Plus 6.0 software; Media Cybernetics Inc, Rockville, MD, USA). Data was only collected in the mornings (9:30 am– 12:30 pm).

**Fig 1:**
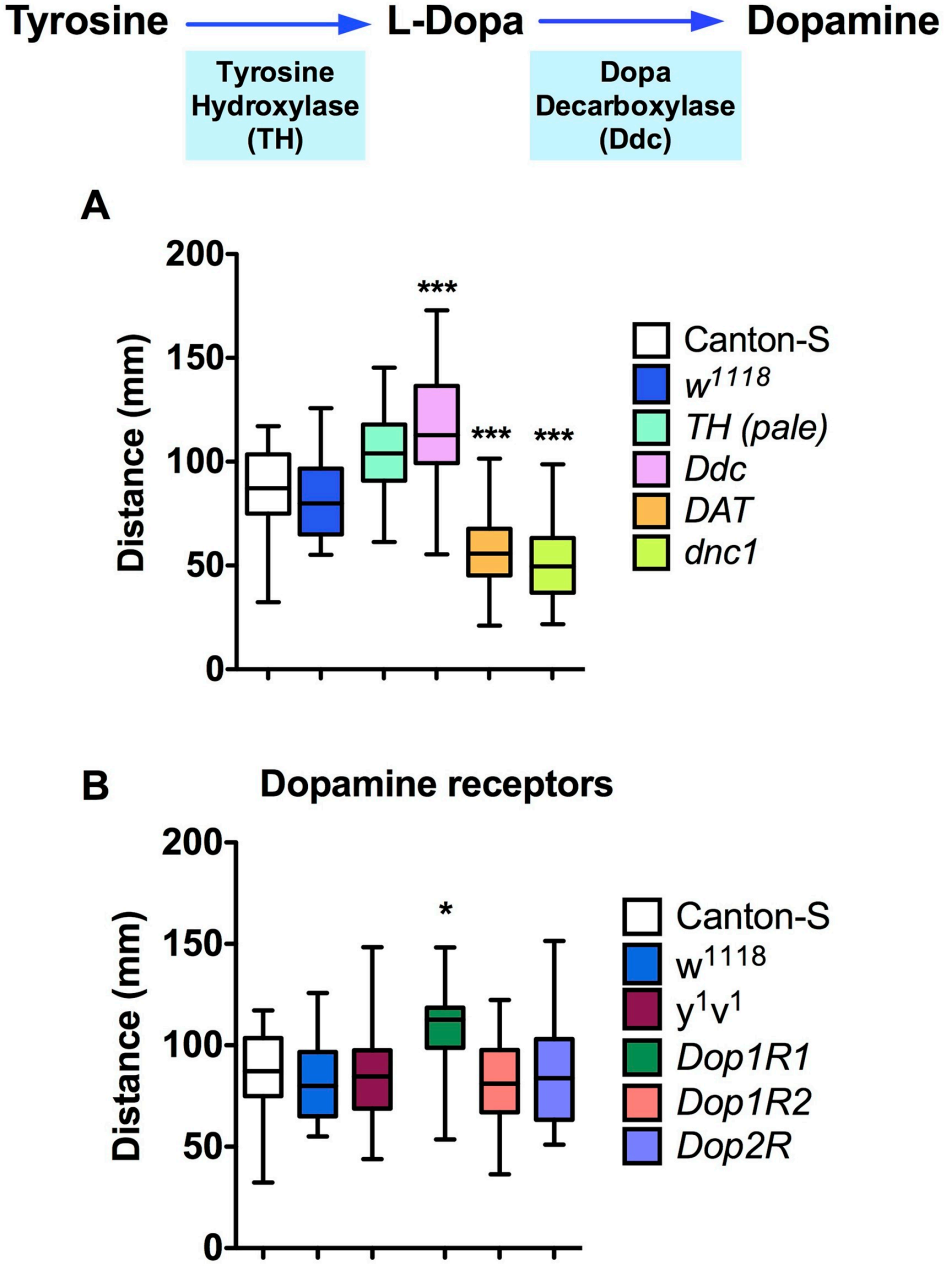
Larvae bearing mutations affecting the dopaminergic system show altered locomotion. A. Motor behavior (distance covered by larvae over 140 sec) was assessed in mutants for the enzymes responsible for dopamine biosynthesis (TH and Ddc), in animals bearing a mutation in the dopamine plasma membrane transporter (DAT), and in larvae mutant for the cAMP-phosphodiesterase (dnc^1^). In the upper insert is indicated the dopamine biosynthetic pathway. B. Motor behavior was assessed in animals containing a mutation for three DA receptors Dop1R1, Dop1R2 and Dop2R. Data shown as a box and whiskers plot from 30 or more animals per genotype. *P<0,05; ***P<0,005, one-way ANOVA followed by Tukey post-hoc test, as compared to controls. Genetic background does not affect motor output in our setup.

**Fig 2:**
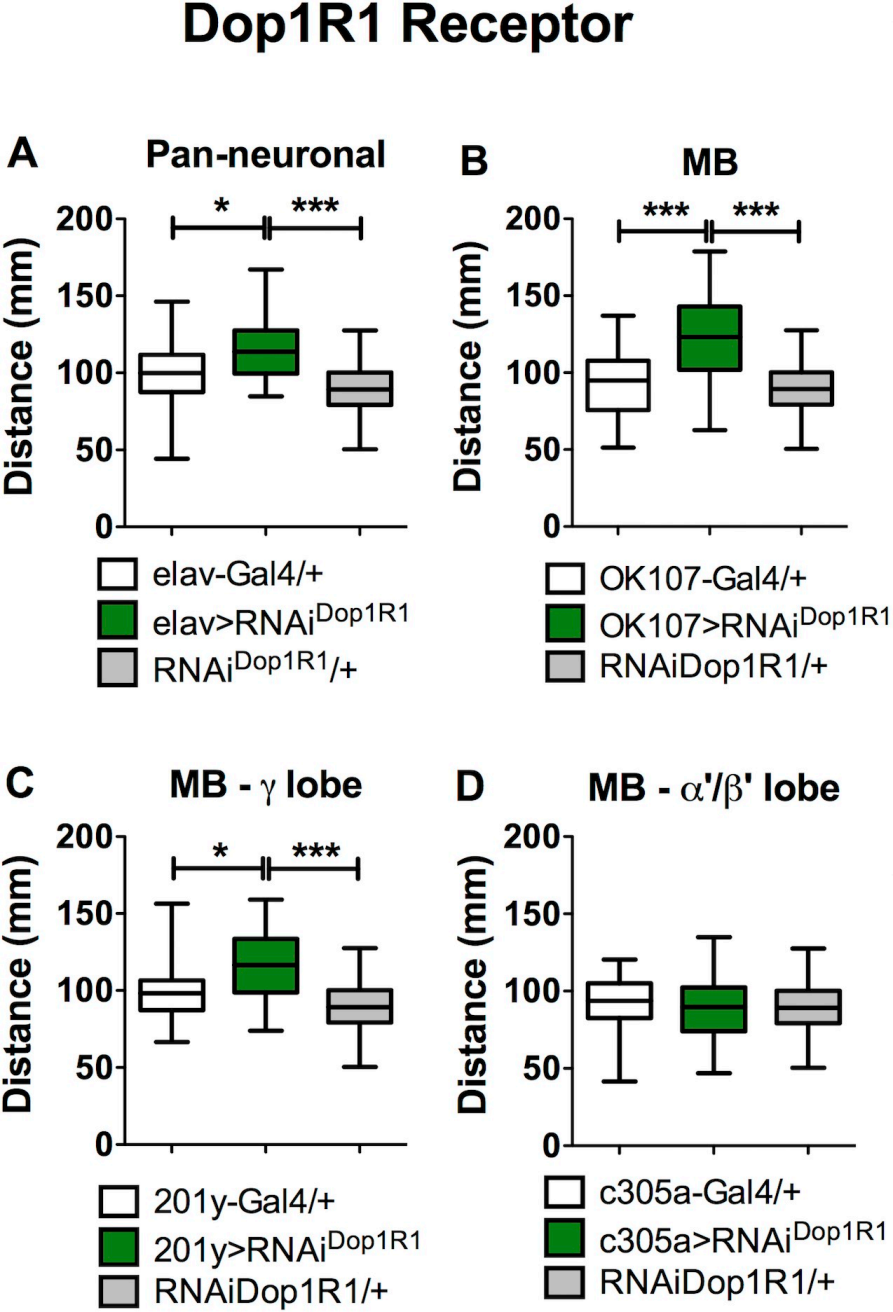
Dop1R1 receptor expressed in MB modulates larval locomotion. A. Pan-neuronal expression of an RNAi against Dop1R1 (elav>RNAi^Dop1R1^) results in increased motor behavior as compared to genetic controls. B. Expression of RNAi^Dop1R1^ in the entire MB neuronal population (OK107>RNAi^Dop1R1^) or C. in the γ-lobe forming neurons (201y>RNAi^Dop1R1^), induces an increase in larval locomotion. D. No effect on locomotion is observed when the RNAi is expressed in the α′β′ MB forming neurons (c305a>RNAi^Dop1R1^). In each case, genetic controls are animals bearing one copy of the Gal4 driver or the undriven UAS-RNAi Dop1R1 (in white and gray bars respectively). Data represent results obtained from at least 34 different larvae per experimental condition. * and *** indicates p<0.05 and p<0.005 compared to genetic controls; one-way ANOVA followed by Tukey post-hoc test.

**Fig 3:**
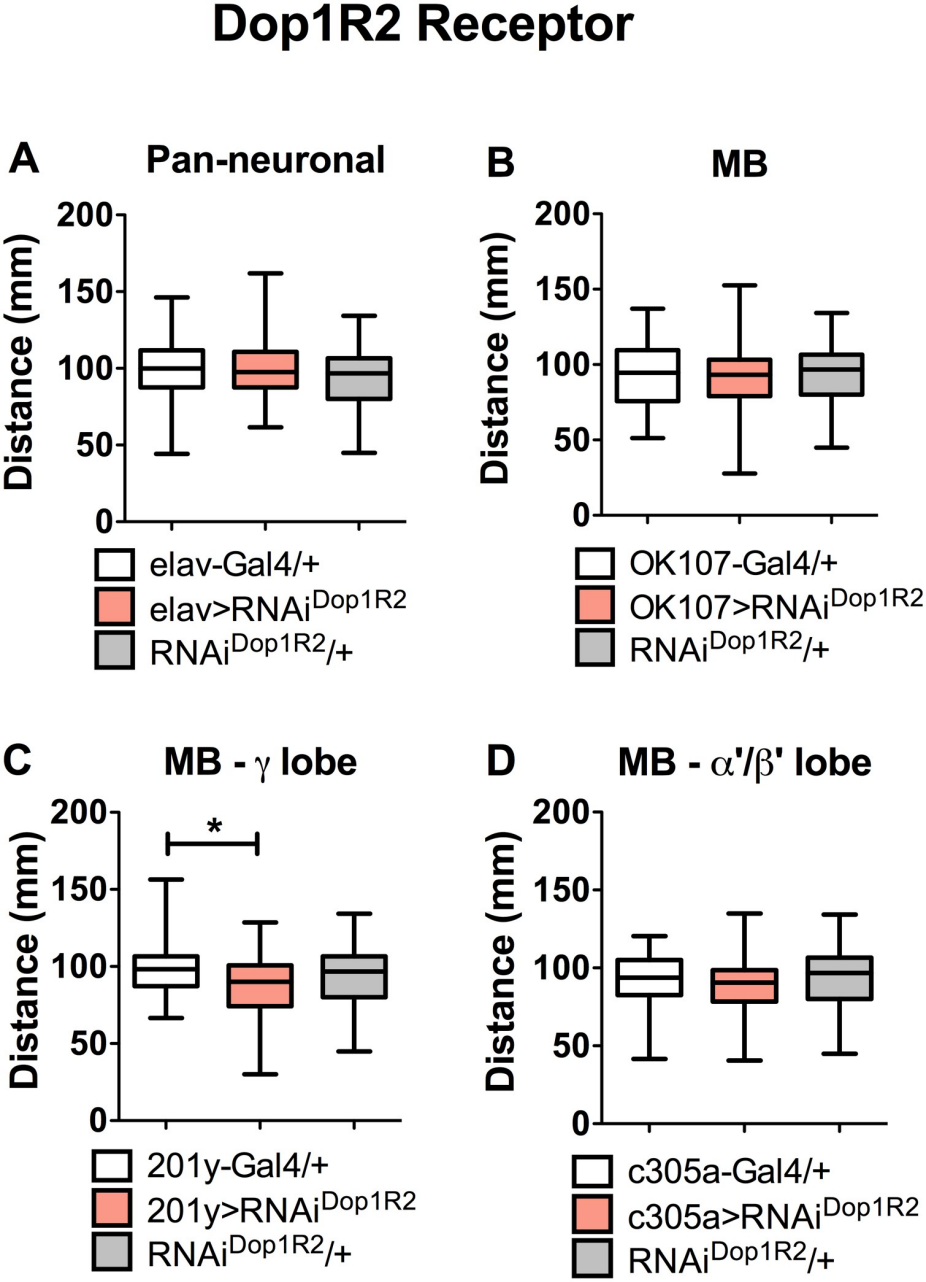
Dop1R2 does not affect larval locomotion. A. Expression of RNAi directed to Dop1R2 transcript (RNAi^Dop1R2^) in the entire CNS (elav>RNAi^Dop1R2^); B. in the MB (OK107>RNAi^Dop1R2^); C. in the MB γ-lobe (201y>RNAi^Dop1R2^); or α′β′ (c305a>RNAi^Dop1R2^) neuronal populations do not affect larval locomotion as compared to genetic controls. Results from 34 larvae or more, per experimental condition. One-way ANOVA followed by Tukey post-hoc test indicated no statistical differences between experimental groups and genetic controls (Gal4/+, white bars; UAS-RNAi^Dop1R2^/+, gray bars), except when indicated (*, p<0.05).

**Fig 4:**
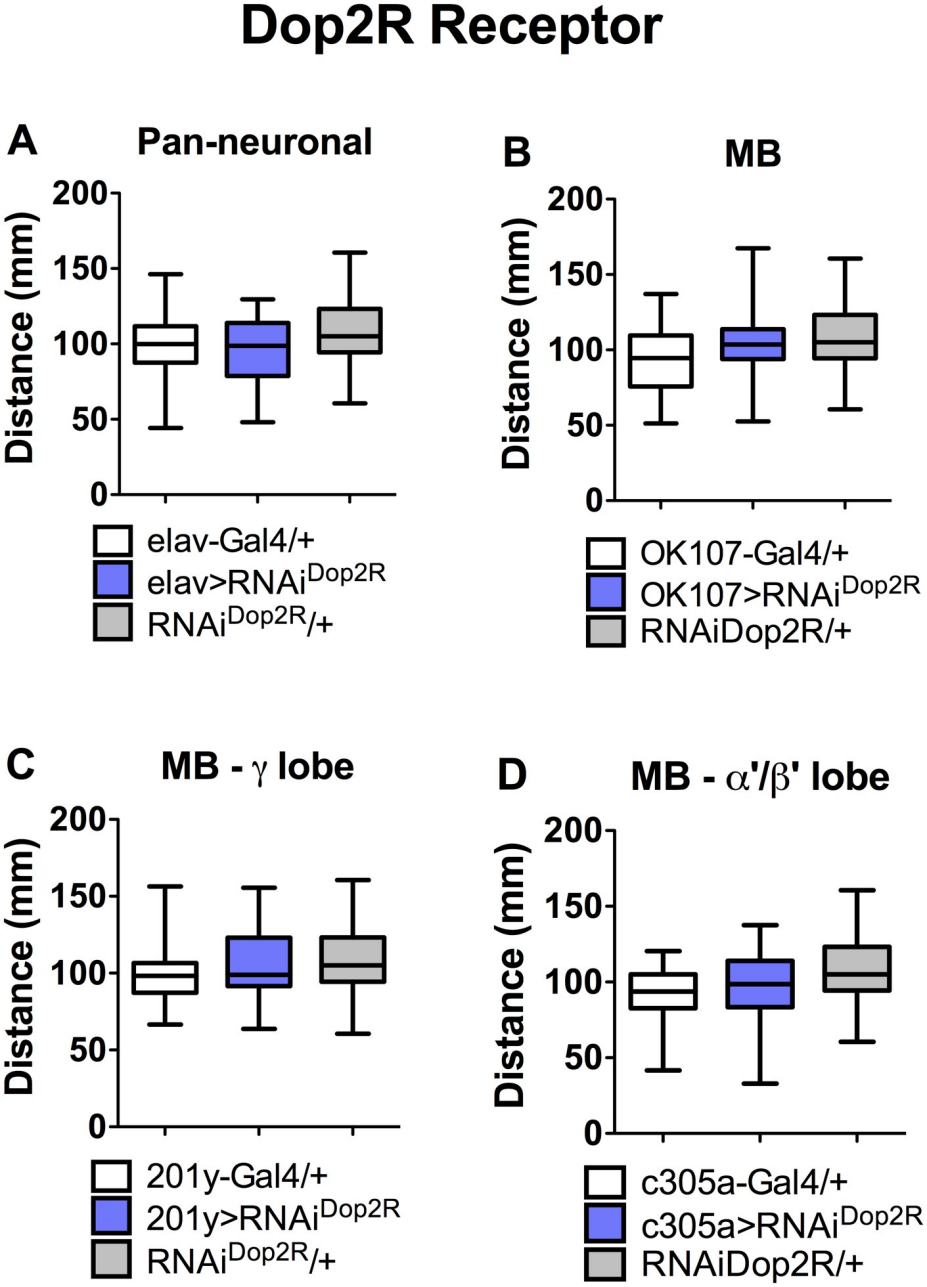
Dop2R does not affect larval locomotion. Expression of an RNAi against Dop2R (RNAi^Dop2R^) A. in the entire CNS (elav>RNAi^Dop2R^); B. in the whole MB (OK107>RNAi^Dop1R2^); or in the neuronal populations forming the C. γ lobe (201y>RNAi^Dop1R2^) or D. α′β′ lobe (c305a>RNAi^Dop1R2^) does not affect locomotion, when compared to genetic controls (Gal4/+, white bars; UAS-RNAi^Dop2R^/+, gray bars). Data obtained from at least 34 larvae per experimental condition. One-way ANOVA followed by Tukey post-hoc test indicated no statistical differences between experimental groups.

**Fig 5:**
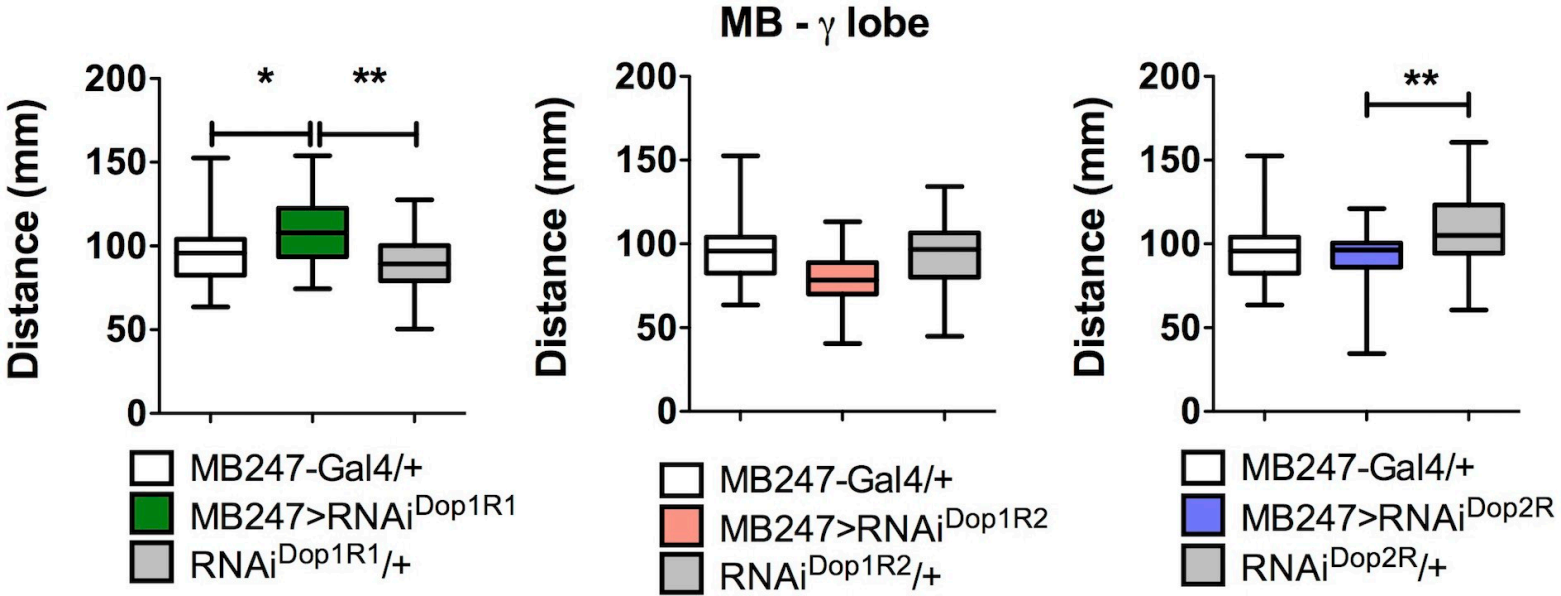
Expression of RNAi^Dop1R1^ in the γ-lobe MB neurons affects motor output in *Drosophila* larvae. Expression of RNAi for Dop1R1, Dop1R2 and Dop2R receptors in MB γ-lobe neurons using a different Gal4 driver line (MB247-Gal4) induces a differential response on motor output. The different RNAi were expressed under the control of this driver that only labels neurons in flies at the larval stage. It is only observed an effect on motor behavior after expression of the RNAi^Dop1R1^ (left panel, MB247>RNAi^Dop1R1^) while no effect is observed after expression of the other RNAis (MB247>RNAi^Dop1R2^ and MB247>RNAi^Dop2R^; center and right panel, respectively) in this neuronal population. These data further support the proposition that the Dop1R1 receptor expressed in the MB γ-lobe is responsible for the effects on locomotion. Data obtained from at least 30 different animals. *, ** indicates p<0.05 and p<0.01, respectively; one-way ANOVA followed by Tukey post-test, when experimental group is compared to respective controls (Gal4/+, in white; undriven UAS-RNAi/+, in gray).

We carry out mating schemes so that experiments with most of the strains are carried out in specific days, in the time window indicated above, to minimize variability explained by environmental factors (fly food, eventual external noises, etc). Thus, all data presented in Fig 1 was recorded in 4 specific experimental days; all data presented in Figs 2–4 was obtained in 6 specific experimental days; data in Fig 5 was obtained in 4 specific experimental days. This explains why in different graphs we use data from a single set of experiments (e.g. in Figs 2A, 3A and 4A, data for “elav-gal4/+” group comes from the same experiment; a similar situation occurs with data for other gal4/+ strains).

### Fast Scan Cyclic Voltammetry (FSCV)

*Ex-vivo* brain recordings were performed as in [29–32], with the carbon fiber electrode placed on top of the AL region. Recordings were carried out in Hepes-Tyrode solution at room temperature, as previously described. A triangle waveform was ramped from −0.4 to 1.2 V and back (vs Ag/AgCl reference electrode) at a scan rate of 400 V/s, every 100 ms (Chem-Clamp potensiostat; Dagan Corporation, Minneapolis, MN, USA). For data collection, two National Instruments acquisition cards (NI-DAQ; PCI-6711 and PCI-6052e; National Instruments, Austin, TX, USA) were used to interface the potentiostat and stimulator with Demon Voltammetry and Analysis software. Brains from wildtype and DAT mutants were exposed to a puff of dopamine (100 μM) in this buffer solution. The time taken to reach 50% of maximum signal after amine application (t_50_) was used as a parameter to evaluate operation of the transporter in DAT mutant.

### Statistics

Statistical analyses were performed using GraphPad Prism 6.0 software (GraphPad Software, La Jolla, CA, USA). Execution of motor programs, evaluated as distance covered over the recorded time, was measured in mm [17]. Data are graphed as box and whiskers plots, where whiskers indicate the lowest and highest measurement per group. One-way ANOVA analysis followed by Tukey post-hoc test was used, unless mentioned otherwise. When expressing RNAi for any given receptor, effects on locomotion were considered significant when statistical analysis showed differences of experimental group as compared to both genetic controls.

All data and statistical analysis presented in this work is available at (DOI: 10.6084/m9.figshare.11823444).

### Biosafety issues

The experimental procedures were approved by the Bioethics and Biosafety Committee of the Facultad de Ciencias Biológicas, Pontificia Universidad Católica de Chile and were conducted in accordance with the guidelines of the National Fund for Scientific and Technological Research (FONDECYT) and the Servicio Agrícola y Ganadero de Chile (SAG).

## Results

### Dopaminergic system contributes to larval locomotion

As a first approach to evaluate whether dopaminergic systems affects motor programs in *Drosophila* larvae, we assessed locomotion in animals expressing mutations for biosynthetic enzymes for dopamine and their receptors (Fig 1). Flies expressing a mutation for the dopamine biosynthetic rate-limiting enzyme, Tyrosine Hydroxylase (TH), shows no statistically difference in locomotion compared to control strains. However, animals expressing a mutation in Dopa decarboxylase (Ddc), an enzyme that contributes in the biosynthesis of both, dopamine and serotonin, show increased locomotion (Fig 1A). This data would be consistent with the idea that dopamine neural systems play a more secondary role in *Drosophila* larval motor control as compared to serotonin, as previously suggested [17]. To further assess the contribution of the dopaminergic system to larval locomotion, we studied motor behavior in mutants for the dopamine transporter, DAT. This protein is a specific component of the dopaminergic neural system, and it has been shown that impairment in DAT results in increased dopaminergic signaling [33]. To demonstrate that the DAT hypomorphic mutant exhibits an impaired reuptake of extracellular dopamine, we exposed brains from DAT mutants and wildtype animals to a puff of dopamine and recorded the electrochemical signals in a FSCV set up. Results show an increase in t_50_ in mutants as compared to wildtype animals (31.65 ± 3.6 and 16.89 ± 2.8 seconds, in mutant and control brains, respectively; p<0.01, t-test), consistent with impaired DAT activity.

Results obtained from larvae mutants for DAT show decreased motor output (Fig 1A). Since the activation of dopaminergic receptors is associated to the modulation of intracellular cAMP levels, we also evaluated locomotion in animals mutant for the enzyme responsible for the degradation of cAMP (the cAMP-phosphodiesterase, dnc^1^). Data obtained show reduced locomotion in dnc^1^ mutant animals (Fig 1A), consistent with the idea that an increased cAMP signaling is associated to reduced larval locomotion.

We then decided to evaluate the contribution of the different dopaminergic receptors to larval motor programs. Several receptors for dopamine have been described (Table 1), but we decided to focus our attention only on those that share homology with vertebrate receptors. Results show that mutant animals for the Dop1R1 receptor show increased motor output, while animals bearing a mutation for Dop1R2 and Dop2R receptors display normal locomotion, as compared to control animals (Fig 1B).

Overall, these results suggest that mutations affecting BA systems differentially modulate larval locomotion and that the protein mediating the effect of dopamine on locomotion is the Dop1R1. However, these data do not indicate what is the region in the larval brain where dopamine is acting.

### The Dop1R1 dopamine receptor in MB inhibits *Drosophila* larval locomotion

The influence of MB on locomotion has been documented mainly in the adult fly [6, 34, 35]. Several reports have argued that the larval MB might also play a role in the modulation of motor programs [13, 17]. The MB is a brain region that receives diverse aminergic innervation and we previously showed that serotonin receptors expressed in MB differentially modulate motor programs in larvae [17]. We decided to assess the proposition that Dop1R1, Dop1R2 and/or Dop2R in MB are responsible for the modulation of motor programs in larvae.

We detected an increase in locomotion in larvae expressing the RNAi^Dop1R1^ pan-neuronally, when compared to controls (Fig 2A). The expression of the RNAi^Dop1R1^ under the control of a gal4 driver for the whole MB, also show increased locomotion (Fig 2B). This suggests that actions of the Dop1R1 receptor on MB neurons would partially explain the effects on locomotion observed in animals expressing pan-neuronally the RNAi^Dop1R1^.

Anatomical and structural studies show that the MB neurons in the adult brain are classified according to their axon projections into α′β′, αβ and γ lobes [36, 37]. The literature on olfactory learning and memory shows that this organization has functional implications: the different lobes are required in different memory phases [38, 39]. It has become evident that the organization of MB in different lobes plays a role in adult fly locomotion, as well [40, 41]. As in the adult fly, it is possible to identify different MB neuronal subpopulations in the larval brain: the γ and the α′β′ lobe neuronal populations [25, 37]. We asked whether these two subpopulations differentially contribute to larval motor output. Animals where expression of the RNAi^Dop1R1^ is directed to the larval γ-lobe MB neurons show increased locomotion, as compared to control animals (Fig 2C). No effect is observed when the RNAi is expressed in the other MB neuronal subpopulation, the α′β′ lobe neurons (Fig 2D).

Interestingly, no effect on locomotion was observed when RNAi for the other dopaminergic receptors were expressed pan-neuronally or specifically in MB neurons (Figs 3 and 4), which is consistent with data obtained with mutant animals for these receptors (Fig 1).

To further validate our results for the Dop1R1, we used another Gal4 driver to direct the expression of the RNAi for the different dopamine receptors in γ lobe neurons. We observed the same effect: only the expression of the RNAi^Dop1R1^ in larval MB γ lobe neurons affects larval locomotion (Fig 5).

Thus, all the data obtained using the different RNAi for dopamine receptors are consistent with results obtained when evaluating motor output in larvae bearing a mutation for the different dopaminergic receptors, and are consistent with the idea that dopaminergic systems innervating MB neurons exert their action on motor programs via Dop1R1.

## Discussion

The MB is considered an association area involved in the generation of olfactory memories in the fly brain [42, 43]. However, it is also involved in other tasks, including the modulation of motor programs. Actually, several reports support the proposition that MB inhibit motor programs in adult flies [6, 34]. On the other hand, little we know on the contribution of MB to motor control in *Drosophila* larvae. We decided to advance our understanding on this issue.

Different aminergic systems modulate motor output in *Drosophila* larvae, including octopamine/tyramine [44]; serotonin [19]. The mechanisms underlying this effect are not fully understood. However, it has been shown that different receptors for amines are expressed in the MB [21, 22, 45]. Thus, we have previously proposed that aminergic systems innervating MB modulate the activity of MB neurons, and consequently, the execution of motor programs. In this regard we previously showed that serotonergic neural systems acting on specific receptors in MB inhibit motor programs in fly larvae [17].

Several evidences support the proposition that dopaminergic systems could contribute to the execution of motor programs in *Drosophila*, although the literature is mainly focused at the adult stage and usually in response to different kind of stimuli [41]. For instance, it has been reported that adult flies expressing a mutation in the TH gene show impaired locomotion [46]. In addition, it has been shown that optogenetic activation of DA neurons increases locomotion [47]. As in the adult brain, larval DA cells innervate the MB region [20]. However, no data is available on the contribution of dopaminergic systems and/or specific receptors to motor programs in larvae. We decided to assess the effect of DA receptors on larval locomotion.

Our data show increased locomotion in animals mutant for *ddc*, the second enzyme in dopamine biosynthesis, and in larvae bearing a mutation in the type1 dopamine receptor Dop1R1. These can be seen as genetic manipulations that result in impaired dopamine signaling: either reduced dopamine biosynthesis or impaired ability to receive the signal to generate a postsynaptic response. On the other hand, animals mutant for DAT, a genetic manipulation associated to increased dopamine extracellular levels [33], exhibit reduced motor output. Thus, dopamine signaling seems to be associated to reduced locomotion in *Drosophila* larvae. Interestingly, activation of D1-type receptor is usually associated to increase in cAMP intracellular levels [22]. Thus, we proposed that animals where increased cAMP intracellular levels are reported should display reduced locomotion. This is what we observe when locomotion is studied in animals mutant for the cAMP-phosphodiesterase (*dnc*^*1*^).

One puzzling observation is the lack of effects of a TH mutation on locomotion. We observed a tendency to increased locomotion in mutants for this enzyme that does not reach statistical significance. Interestingly, in a previous work it was argued that dopamine play different roles in several behaviors [48]. Actually, in that work it is argued that the contribution of dopamine is more important in the planning and execution of exploratory behaviors, than in other motor behaviors. Thus, it is possible to propose that different larval behaviors are differentially affected by dopamine deficiency. On the other hand, mutants on the second enzyme in dopamine biosynthesis exhibit increased motor output. This enzyme also contributes to serotonin biosynthesis and we have previously shown that this amine strongly contributes to larval locomotion [17]. Thus, the robust effect on locomotion observed in *ddc* mutants would be the consequence of the two aminergic systems affected: dopaminergic and serotoninergic.

Next, we continued evaluating the contribution of dopaminergic receptors on locomotion. Consistent with the idea that Dop1R1 expressed in CNS neurons affects motor programs, pan-neuronal expression of an RNAi directed to this receptor results in increased motor output. No effect was observed in mutants or animals expressing RNAi for the other DA receptors. Thus, our data suggest that DA neural systems acting on Dop1R1 inhibit the execution of motor programs. Further experiments demonstrate that at least one of the brain regions where this receptor is acting to modulate locomotion is the larval MB, particularly the γ lobe neurons.

Previous reports have shown the expression of Dop1R1 receptor in both the adult and larval MB [22] and its relevance to olfactory learning and memory in adult flies [49, 50]. Likewise, it has been reported an association between this receptor and different behaviors including sleep homeostasis and short and long lasting memories in adult flies [51–53]. Interestingly, it has been also suggested that the Dop1R1 expressed in MB is involved in motor-related behaviors in adult flies, in particular, the arousing effects of caffeine and amphetamine [54]. Overall, these data support the idea that Dop1R1 expressed in MB would be essential in the modulation of behaviors both in adult flies and larvae.

It has been previously shown the expression of the other type 1 DA receptor, Dop1R2, in *Drosophila* larval MB [21]. The fact that our results suggest this receptor is not involved in the execution of larval motor programs is rather intriguing. However, immunohistochemistry studies have shown Dop1R2 is expressed mostly in the α’/β’ MB lobes [21] while the Dop1R1 is found in the γ lobe [22]. Therefore, the differential effects of these two D1-type DA receptors would be explained by their differential expression in the MB region.

The other DA receptor of interest, the type 2 Dop2R, has been associated to the control of locomotion in adult flies. However, this receptor is not expressed in MB neurons, either in larvae or in adult flies [20]. Thus, the effect of Dop2R on locomotion seems to be explained by actions of this receptor in regions different from the MB. Remarkably, the Dop2R is proposed to be localized in axon terminals, acting as a presynaptic receptor to modulate the release of DA, as in mammals [55, 56]. Thus, it seems plausible to propose that a modification in the release of amines (dopamine) in the MB region could indirectly modulate the execution of motor programs in adult flies and/or in larvae. Some of the new tools recently generated by different research groups [29, 30, 55] would be useful to evaluate this idea.

**Fig 6:**
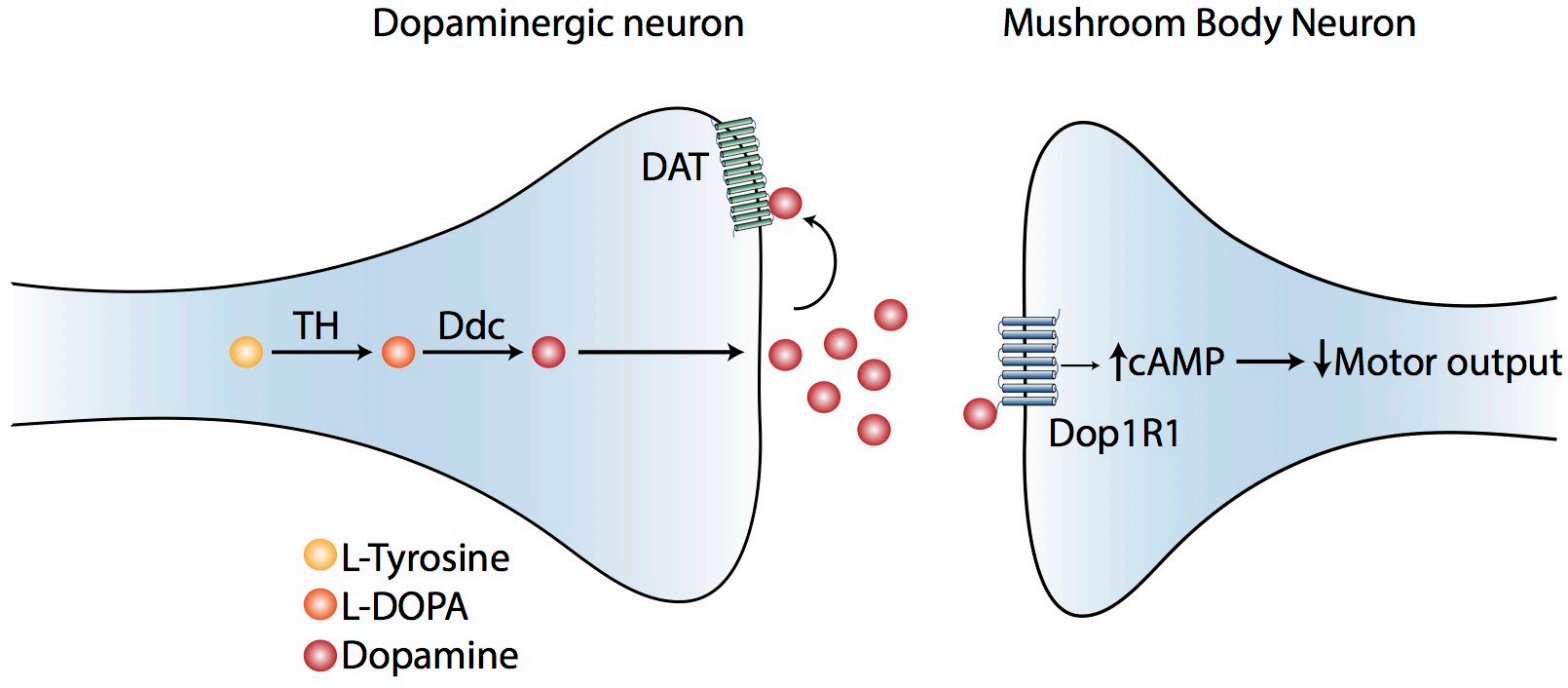
A model to explain the contribution of Dop1R1 expressed in MB to *Drosophila* larval locomotion. This model proposes, that the type 1 Dop1R1 receptor is expressed in MB and activated by dopaminergic neurons arriving to this brain area. The activation of the dopamine receptor induces an increase in intracellular cAMP levels, which results in decreased motor output. DAT, the dopamine plasma membrane transporter, regulates the availability of dopamine in the synaptic cleft, so that when it is not present or not functional, increases dopaminergic signaling, which results in reduced motor output.

Overall, our data suggest that dopaminergic systems acting specifically on the γ lobe neurons activate the type-1 dopamine receptor Dop1R1, to modulate the execution of motor programs in *Drosophila* larvae. All the ideas and findings here presented and discussed have been summarized in a working model (Fig 6).

In vertebrates the Striatum is one of the key brain structures involved in the control of motor behavior [57]. Several evidences show the importance of dopamine D1-type receptors in modulating the activity of striatal neurons, a phenomenon that underlies the control of motor output in vertebrates (reviewed in [58, 59]). Our data further support the proposition that at least part of the mechanism regulating behaviors in vertebrates and invertebrates, including motor control, are conserved throughout evolution.

## References

[1] Klein MO, Battagello DS, Cardoso AR, Hauser DN, Bittencourt JC, Correa RG. Dopamine: Functions, Signaling, and Association with Neurological Diseases. Cell Mol Neurobiol. 2019;39(1):31–59. 10.1007/s10571-018-0632-3 30446950 PMC11469830

[2] Meder D, Herz DM, Rowe JB, Lehericy S, Siebner HR. The role of dopamine in the brain—lessons learned from Parkinson's disease. Neuroimage. 2018. 10.1016/j.neuroimage.2018.11.021 30465864

[3] Monastirioti M. Biogenic amine systems in the fruit fly Drosophila melanogaster. Microsc Res Tech. 1999;45(2):106–121. 10.1002/(SICI)1097-0029(19990415)45:2<106::AID-JEMT5>3.0.CO;2-3 10332728

[4] Chang HY, Grygoruk A, Brooks ES, Ackerson LC, Maidment NT, Bainton RJ, et al Overexpression of the Drosophila vesicular monoamine transporter increases motor activity and courtship but decreases the behavioral response to cocaine. Mol Psychiatry. 2006;11(1):99–113. 10.1038/sj.mp.4001742 16189511

[5] Hardie SL, Zhang JX, Hirsh J. Trace amines differentially regulate adult locomotor activity, cocaine sensitivity, and female fertility in Drosophila melanogaster. Dev Neurobiol. 2007;67(10):1396–1405. 10.1002/dneu.20459 17638385

[6] Martin JR, Ernst R, Heisenberg M. Mushroom bodies suppress locomotor activity in Drosophila melanogaster. Learn Memory. 1998;5(1–2):179–191. PMC311252 10454382

[7] Strauss R. The central complex and the genetic dissection of locomotor behaviour. Curr Opin Neurobiol. 2002;12(6):633–638. 10.1016/s0959-4388(02)00385-9 12490252

[8] Strauss R, Heisenberg M. A Higher Control Center of Locomotor Behavior in the Drosophila Brain. J Neurosci. 1993;13(5):1852–1861. 10.1523/JNEUROSCI.13-05-01852.1993 8478679 PMC6576564

[9] Kahsai L, Carlsson MA, Winther AME, Nassel DR. Distribution of Metabotropic Receptors of Serotonin, Dopamine, Gaba, Glutamate, and Short Neuropeptide F in the Central Complex of Drosophila. Neuroscience. 2012;208:11–26. 10.1016/j.neuroscience.2012.02.007 22361394

[10] Kong EC, Woo K, Li HY, Lebestky T, Mayer N, Sniffen MR, et al A Pair of Dopamine Neurons Target the D1-Like Dopamine Receptor DopR in the Central Complex to Promote Ethanol-Stimulated Locomotion in Drosophila. Plos One. 2010;5(3). 10.1371/journal.pone.0009954 PMC2848596 20376353

[11] Lebestky T, Chang JSC, Dankert H, Zelnik L, Kim YC, Han KA, et al Two Different Forms of Arousal in Drosophila Are Oppositely Regulated by the Dopamine D1 Receptor Ortholog DopR via Distinct Neural Circuits. Neuron. 2009;64(4):522–536. 10.1016/j.neuron.2009.09.031 19945394 PMC2908595

[12] Pech U, Pooryasin A, Birman S, Fiala A. Localization of the Contacts Between Kenyon Cells and Aminergic Neurons in the Drosophila melanogaster Brain Using SplitGFP Reconstitution. J Comp Neurol. 2013;521(17):3992–4026. 10.1002/cne.23388 23784863

[13] Scantlebury N, Zhao XL, Rodriguez Moncalvo VG, Camiletti A, Zahanova S, Dineen A, et al The Drosophila gene RanBPM functions in the mushroom body to regulate larval behavior. PLoS One. 2010;5(5):e10652 10.1371/journal.pone.0010652 20498842 PMC2871054

[14] Varnam CJ, Strauss R, DeBelle JS, Sokolowski MB. Larval behavior of Drosophila central complex mutants: Interactions between no bridge, foraging, and chaser. J Neurogenet. 1996;11(1–2):99–115. 10.3109/01677069609107065 10876652

[15] Huser A, Rohwedder A, Apostolopoulou AA, Widmann A, Pfitzenmaier JE, Maiolo EM, et al The Serotonergic Central Nervous System of the Drosophila Larva: Anatomy and Behavioral Function. Plos One. 2012;7(10). 10.1371/journal.pone.0047518 PMC3474743 23082175

[16] Selcho M, Pauls D, Han KA, Stocker RF, Thum AS. The role of dopamine in Drosophila larval classical olfactory conditioning. PLoS One. 2009;4(6):e5897 10.1371/journal.pone.0005897 19521527 PMC2690826

[17] Silva B, Goles NI, Varas R, Campusano JM. Serotonin receptors expressed in Drosophila mushroom bodies differentially modulate larval locomotion. PLoS One. 2014;9(2):e89641 10.1371/journal.pone.0089641 24586928 PMC3934909

[18] Suster ML, Martin JR, Sung C, Robinow S. Targeted expression of tetanus toxin reveals sets of neurons involved in larval locomotion in Drosophila. J Neurobiol. 2003;55(2):233–246. 10.1002/neu.10202 12672020

[19] Moncalvo VGR, Campos AR. Role of serotonergic neurons in the Drosophila larval response to light. Bmc Neurosci. 2009;10. 10.1186/1471-2202-10-66 PMC2711092 19549295

[20] Draper I, Kurshan PT, McBride E, Jackson FR, Kopin AS. Locomotor activity is regulated by D2-like receptors in Drosophila: an anatomic and functional analysis. Dev Neurobiol. 2007;67(3):378–393. 10.1002/dneu.20355 17443795

[21] Han KA, Millar NS, Grotewiel MS, Davis RL. DAMB, a novel dopamine receptor expressed specifically in Drosophila mushroom bodies. Neuron. 1996;16(6):1127–1135. 10.1016/s0896-6273(00)80139-7 8663989

[22] Kim YC, Lee HG, Seong CS, Han KA. Expression of a D1 dopamine receptor dDA1/DmDOP1 in the central nervous system of Drosophila melanogaster. Gene Expr Patterns. 2003;3(2):237–245. 10.1016/s1567-133x(02)00098-4 12711555

[23] Srivastava DP, Yu EJ, Kennedy K, Chatwin H, Reale V, Hamon M, et al Rapid, nongenomic responses to ecdysteroids and catecholamines mediated by a novel Drosophila G-protein-coupled receptor. J Neurosci. 2005;25(26):6145–6155. 10.1523/JNEUROSCI.1005-05.2005 15987944 PMC6725065

[24] Duffy JB. GAL4 system in Drosophila: a fly geneticist's Swiss army knife. Genesis. 2002;34(1–2):1–15. 10.1002/gene.10150 12324939

[25] Pauls D, Selcho M, Gendre N, Stocker RF, Thum AS. Drosophila larvae establish appetitive olfactory memories via mushroom body neurons of embryonic origin. J Neurosci. 2010;30(32):10655–10666. 10.1523/JNEUROSCI.1281-10.2010 20702697 PMC6634688

[26] Inagaki HK, Ben-Tabou de-Leon S, Wong AM, Jagadish S, Ishimoto H, Barnea G, et al Visualizing neuromodulation in vivo: TANGO-mapping of dopamine signaling reveals appetite control of sugar sensing. Cell. 2012;148(3):583–595. 10.1016/j.cell.2011.12.022 22304923 PMC3295637

[27] Liu QL, Liu S, Kodama L, Driscoll MR, Wu MN. Two Dopaminergic Neurons Signal to the Dorsal Fan-Shaped Body to Promote Wakefulness in Drosophila. Current Biology. 2012;22(22):2114–2123. 10.1016/j.cub.2012.09.008 23022067 PMC3505250

[28] Neckameyer WS, Bhatt P. Neurotrophic actions of dopamine on the development of a serotonergic feeding circuit in Drosophila melanogaster. Bmc Neurosci. 2012;13:26 10.1186/1471-2202-13-26 22413901 PMC3364880

[29] Fuenzalida-Uribe N, Meza RC, Hoffmann HA, Varas R, Campusano JM. nAChR-induced octopamine release mediates the effect of nicotine on a startle response in Drosophila melanogaster. J Neurochem. 2013;125(2):281–290. 10.1111/jnc.12161 23331098

[30] Fuenzalida-Uribe N, Hidalgo S, Varas R, Campusano JM. Study of the contribution of Nicotinic Receptors to the release of endogenous Biogenic amines in Drosophila brain In Nicotinic Acetylcholine Receptor Technologies. [S.l.]: Springer Science+Business Media New York; 2016 http://SK8ES4MC2L.search.serialssolutions.com/?sid=sersol&SS_jc=TC_025758082&title=Nicotinic%20Acetylcholine%20Receptor%20Technologies.

[31] Hidalgo S, Molina-Mateo D, Escobedo P, Zarate RV, Fritz E, Fierro A, et al Characterization of a Novel Drosophila SERT Mutant: Insights on the Contribution of the Serotonin Neural System to Behaviors. ACS Chem Neurosci. 2017;8(10):2168–2179. 10.1021/acschemneuro.7b00089 28665105

[32] Molina-Mateo D, Fuenzalida-Uribe N, Hidalgo S, Molina-Fernandez C, Abarca J, Zarate RV, et al Characterization of a presymptomatic stage in a Drosophila Parkinson's disease model: Unveiling dopaminergic compensatory mechanisms. Biochim Biophys Acta Mol Basis Dis. 2017;1863(11):2882–2890. 10.1016/j.bbadis.2017.07.013 28716706

[33] Vickrey TL, Xiao N, Venton BJ. Kinetics of the dopamine transporter in Drosophila larva. ACS Chem Neurosci. 2013;4(5):832–837. 10.1021/cn400019q 23600464 PMC3656763

[34] Helfrich-Forster C, Wulf J, de Belle JS. Mushroom body influence on locomotor activity and circadian rhythms in Drosophila melanogaster. J Neurogenet. 2002;16(2):73–109. 10.1080/01677060213158 12479377

[35] Xiong Y, Lv H, Gong Z, Liu L. Fixation and locomotor activity are impaired by inducing tetanus toxin expression in adult Drosophila brain. Fly (Austin). 2010;4(3):194–203. 20657190 10.4161/fly.12668

[36] Crittenden JR, Skoulakis EM, Han KA, Kalderon D, Davis RL. Tripartite mushroom body architecture revealed by antigenic markers. Learn Mem. 1998;5(1–2):38–51. 10454371 PMC311260

[37] Lee T, Lee A, Luo L. Development of the Drosophila mushroom bodies: sequential generation of three distinct types of neurons from a neuroblast. Development. 1999;126(18):4065–4076. 10457015 10.1242/dev.126.18.4065

[38] McGuire SE, Deshazer M, Davis RL. Thirty years of olfactory learning and memory research in Drosophila melanogaster. Prog Neurobiol. 2005;76(5):328–347. 10.1016/j.pneurobio.2005.09.003 16266778

[39] Isabel G, Pascual A, Preat T. Exclusive consolidated memory phases in Drosophila. Science. 2004;304(5673):1024–1027. 10.1126/science.1094932 15143285

[40] Riemensperger T, Issa AR, Pech U, Coulom H, Nguyen MV, Cassar M, et al A single dopamine pathway underlies progressive locomotor deficits in a Drosophila model of Parkinson disease. Cell Rep. 2013;5(4):952–960. 10.1016/j.celrep.2013.10.032 24239353

[41] Sun J, Xu AQ, Giraud J, Poppinga H, Riemensperger T, Fiala A, et al Neural Control of Startle-Induced Locomotion by the Mushroom Bodies and Associated Neurons in Drosophila. Front Syst Neurosci. 2018;12:6 10.3389/fnsys.2018.00006 29643770 PMC5882849

[42] Dubnau J, Grady L, Kitamoto T, Tully T. Disruption of neurotransmission in Drosophila mushroom body blocks retrieval but not acquisition of memory. Nature. 2001;411(6836):476–480. 10.1038/35078077 11373680

[43] McGuire SE, Le PT, Davis RL. The role of Drosophila mushroom body signaling in olfactory memory. Science. 2001;293(5533):1330–1333. 10.1126/science.1062622 11397912

[44] Selcho M, Pauls D, El Jundi B, Stocker RF, Thum AS. The role of octopamine and tyramine in Drosophila larval locomotion. J Comp Neurol. 2012;520(16):3764–3785. 10.1002/cne.23152 22627970

[45] Blenau W, Thamm M. Distribution of serotonin (5-HT) and its receptors in the insect brain with focus on the mushroom bodies: lessons from Drosophila melanogaster and Apis mellifera. Arthropod Struct Dev. 2011;40(5):381–394. 10.1016/j.asd.2011.01.004 21272662

[46] Riemensperger T, Isabel G, Coulom H, Neuser K, Seugnet L, Kume K, et al Behavioral consequences of dopamine deficiency in the Drosophila central nervous system. Proc Natl Acad Sci U S A. 2011;108(2):834–839. 10.1073/pnas.1010930108 21187381 PMC3021077

[47] Zhang W, Ge W, Wang Z. A toolbox for light control of Drosophila behaviors through Channelrhodopsin 2-mediated photoactivation of targeted neurons. Eur J Neurosci. 2007;26(9):2405–2416. 10.1111/j.1460-9568.2007.05862.x 17970730

[48] Neckameyer WS. Multiple roles for dopamine in Drosophila development. Dev Biol. 1996;176(2):209–219. 10.1006/dbio.1996.0128 8660862

[49] Berry JA, Cervantes-Sandoval I, Nicholas EP, Davis RL. Dopamine is required for learning and forgetting in Drosophila. Neuron. 2012;74(3):530–542. 10.1016/j.neuron.2012.04.007 22578504 PMC4083655

[50] Kim YC, Lee HG, Han KA. Classical reward conditioning in Drosophila melanogaster. Genes Brain Behav. 2007;6(2):201–207. 10.1111/j.1601-183X.2006.00241.x 16740144

[51] Seugnet L, Galvin JE, Suzuki Y, Gottschalk L, Shaw PJ. Persistent short-term memory defects following sleep deprivation in a drosophila model of Parkinson disease. Sleep. 2009;32(8):984–992. 10.1093/sleep/32.8.984 19725249 PMC2717205

[52] Seugnet L, Suzuki Y, Donlea JM, Gottschalk L, Shaw PJ. Sleep deprivation during early-adult development results in long-lasting learning deficits in adult Drosophila. Sleep. 2011;34(2):137–146. 10.1093/sleep/34.2.137 21286249 PMC3022932

[53] Seugnet L, Suzuki Y, Vine L, Gottschalk L, Shaw PJ. D1 receptor activation in the mushroom bodies rescues sleep-loss-induced learning impairments in Drosophila. Curr Biol. 2008;18(15):1110–1117. 10.1016/j.cub.2008.07.028 18674913 PMC2603029

[54] Andretic R, Kim YC, Jones FS, Han KA, Greenspan RJ. Drosophila D1 dopamine receptor mediates caffeine-induced arousal. Proc Natl Acad Sci U S A. 2008;105(51):20392–20397. 10.1073/pnas.0806776105 19074291 PMC2629270

[55] Vickrey TL, Venton BJ. Drosophila Dopamine2-like receptors function as autoreceptors. ACS Chem Neurosci. 2011;2(12):723–729. 10.1021/cn200057k 22308204 PMC3269839

[56] Wiemerslage L, Schultz BJ, Ganguly A, Lee D. Selective degeneration of dopaminergic neurons by MPP(+) and its rescue by D2 autoreceptors in Drosophila primary culture. J Neurochem. 2013;126(4):529–540. 10.1111/jnc.12228 23452092 PMC3737274

[57] Graybiel AM. The basal ganglia. Curr Biol. 2000;10(14):R509–511. 10.1016/s0960-9822(00)00593-5 10899013

[58] Nishi A, Kuroiwa M, Shuto T. Mechanisms for the modulation of dopamine D-1 receptor signaling in striatal neurons. Front Neuroanat. 2011;5. 10.3389/fnana.2011.00043 PMC3140648 21811441

[59] Surmeier DJ, Ding J, Day M, Wang Z, Shen W. D1 and D2 dopamine-receptor modulation of striatal glutamatergic signaling in striatal medium spiny neurons. Trends Neurosci. 2007;30(5):228–235. 10.1016/j.tins.2007.03.008 17408758

